# Adolescent deliveries in urban Cameroon: a retrospective analysis of the prevalence, 6-year trend and adverse outcomes

**DOI:** 10.1186/s13104-018-3578-0

**Published:** 2018-07-13

**Authors:** Rita F. Tamambang, Tsi Njim, Albertine E. Njie, Lawrence Mbuagbaw, Agnès Mafuta, Mesack Tchana, Simeon-Pierre Choukem

**Affiliations:** 1 Department of Internal Medicine and Paediatrics, Faculty of Health Sciences, University of Buea, Buea, Cameroon; 2 Health and Human Development (2HD) Research Network, Douala, Cameroon; 3 Centre for Tropical Medicine and Global Health, Nuffield Department of Medicine, University of Oxford, Oxfordshire, UK; 4 Saint-Albert Legrand Hospital, Douala, Cameroon; 5 Obstetric and Gynecology Unit, Douala Laquintinie Hospital, Douala, Cameroon; 6 Diabetes and Endocrine Unit, Department of Internal Medicine, Douala General Hospital, Douala, Douala, Cameroon

**Keywords:** Adolescent deliveries, Adverse outcome, Douala, Cameroon

## Abstract

**Objective:**

Adolescent deliveries remain a public health problem in most developing countries. The aim of our study was to determine the prevalence, trends and outcome of adolescent deliveries in an urban setting in Cameroon. We carried out a retrospective register analysis over a 6-year period (January 2010–December 2015) at the Saint Albert Le Grand hospital Douala.

**Results:**

The overall prevalence of adolescent deliveries was 8.2% (662 out of 8056). There was a significant decrease over the 6-year period (p-trend: < 0.05). Adolescents were at higher risk of preterm deliveries (gestational age < 37 weeks; odds ratio [OR], 1.7; 95% confidence interval [CI]; 1.3–2.2; p < 0.01): low birth weight (defined as birth weight < 2650 g, OR; 1.7, CI 1.4–2.2, p < 0.01) and asphyxia at 1st minute (OR, 1.5; 95% CI 1.1–2.2; p = 0.02). There was no difference in delivery outcomes between early and late adolescents. Our results suggest that the prevalence of adolescent deliveries is lower in urban settings. Adolescent deliveries are more likely to result in adverse fetal outcomes than adult deliveries. Measures directed towards the prevention of adolescent pregnancies should be implemented to reduce neonatal morbidity and mortality.

**Electronic supplementary material:**

The online version of this article (10.1186/s13104-018-3578-0) contains supplementary material, which is available to authorized users.

## Introduction

Adolescent pregnancy is defined by WHO as pregnancy in girls aged 10–19 years [1]. Adolescent pregnancy constitutes a serious public health problem worldwide [2, 3]. It is estimated that about 11% of births worldwide are due to adolescents aged between 16 and 19 years with more than 90% occurring in low and middle-income countries [1]. Adolescence in females is a transitional phase of physical and mental development between childhood and adulthood [4].

Pregnancies occurring during this period are considered to be at a higher risk [4] due to biological disadvantages associated with childbearing in this age group [5, 6]. The social and economic disadvantages of this age group [6–8] coupled with other adverse behaviours predominant in adolescents such as substance abuse and inconsistent prenatal care increase this risk [9–11]. Previous studies have reported an increase in the following adverse maternal and perinatal outcomes associated with adolescent pregnancies: preeclampsia/eclampsia, operative deliveries, maternal death, low birth weight (LBW), preterm deliveries, still births and neonatal asphyxia [12–14]. Prevention of adolescent pregnancies hence deliveries, could reduce maternal and neonatal morbidity and mortality [15]. However, like most Sub-Saharan African countries, Cameroon has a high prevalence of adolescent pregnancies [12] varying from 6.87 to 26.51% across regions of the country [16].

We recently carried out a study in a sub-urban setting in Cameroon where the prevalence of adolescent delivery was high (9.1%) with no significant change in the annual prevalence for 6 years [13]. As urbanization has been shown to reduce the prevalence of adolescent deliveries [14], we therefore sought to determine the prevalence, trends and outcome of adolescent deliveries in an urban setting in Cameroon.

## Main text

### Materials and methods

We carried out a retrospective file analysis at the Saint Albert Le Grand hospital of Douala (SAGHD). SAGHD is a denominational hospital a located in Bonaberi, Douala. It was ranked the fourth best maternity unit in Cameroon because of its well-kept registers [17]. It carries out about 1000 deliveries per year; more than other health facilities in its environ. It receives referred cases from neighboring hospitals and offers all the services of general medicine but lacks a neonatal intensive care unit (NICU). The maternity unit has a single gynecologist assisted by a nurse-in-charge.

Complete records of newborns delivered at SAGHD over 6 years (1st January 2010–31st December 2015) were reviewed. Records were considered complete if they had the following information: sociodemographic characteristics (maternal age) and obstetric information (gravidity, parity, gestational age, type of delivery, birth weight and Apgar score in the 1st, 5th and 10th min). These data had been collected by the midwives after delivery and securely stored in archives. Records were excluded if they had deliveries born before viability (> 28 weeks of gestation in our setting), twin deliveries or were incomplete. The reasons for exclusion of records in shown in Additional file 1.

### Data collection, variables and measurements

Relevant data collected included: We collected data on the sociodemographic characteristics of the study population (age), clinical characteristics (gestational age and sex of the neonates), maternal outcome (mode of delivery) and foetal outcome (birthweight, first, fifth and tenth minute Apgar scores and term of the pregnancy which was determined from the gestational age).

### Ethical approval

Ethical approval was obtained from the Institutional Ethics Committee for Research in Human Health of the University of Douala, and administrative clearance from the administration of the SAGHD.

### Statistical analysis

Data was analyzed using Epi-info version 7. Categorical variables were summarised as frequencies and percentages. Continuous variables were presented as means and standard deviations. Frequencies were compared using the Fisher’s exact test. Odds ratio with 95% confidence interval were used to describe the association between categorical variables.

### Results

#### General characteristics of participants

A total of 8056 records were reviewed from 10,002 records giving a response rate of 80.5%. The mean age of the mothers was 26.6 ± 6.4 years.

#### Prevalence and trends of adolescent deliveries

Of the 8056 mothers, 662 were aged less than 20 years giving a prevalence of adolescent deliveries of 8.2% (95% CI 7.6–8.8). The prevalence of early (age ≤ 16 years) and late adolescent (17–19 years) deliveries were 0.77% (62 out of 8056) and 7.45% (600 out of 8056), respectively.

There was initially an increase in the trend of adolescent deliveries from 9.1% (137 out of 1504) in the year 2010 to 10.4% (154 out of 1477) in the year 2011 then a progressive decrease to 6.9% (92 out of 1337: p-trend < 0.05) in the year 2015 (Additional file 2). The annual prevalence of early vs late pregnancy is shown in Table 1.

**Table 1:** Annual prevalence of early (age < 16 years) and late (17–19 years) adolescent deliveries

Year	Total number of deliveries per year	Early adolescents deliveries (n = 62)		Late adolescents deliveries (n = 600)	
		n	Prevalence (%)	N	Prevalence (%)
2010	1504	16	1.1	121	8.1
2011	1477	8	0.5	146	9.9
2012	819	10	1.2	73	8.9
2013	1568	8	0.5	92	5.9
2014	1351	7	0.5	89	6.6
2015	1337	13	1.0	79	5.9

#### Outcomes associated with adolescent deliveries

Adolescent deliveries were significantly associated with the following adverse neonatal outcomes: preterm delivery (OR; 1.7, p ≤ 0.01), Neonatal asphyxia at 1st minute (OR; 1.5, p = 0.02) and LBW (OR; 1.7, p ≤ 0.01) (Table 2). In sub-group analysis there was no statistical significant difference in the occurrence of outcomes between early and late adolescents (Table 3).

**Table 2:** Outcome of adolescent deliveries in the Saint Albert Le Grand Hospital from January 2010 to December 2015

Outcomes	Adolescents (N = 662)		Adults (N = 7394)		OR	95% CI	P value
	N	%	N	%			
Asphyxia at 1st minute	38	5.7	281	3.8	1.5	1.1–2.2	0.02
Still birth	8	1.2	99	1.3	0.9	0.4–1.9	1.0
Preterm delivery	81	12.2	553	7.5	1.7	1.3–2.2	< 0.01
Low birth weight	114	17.2	789	10.7	1.7	1.4–2.2	< 0.01
Caesarean section	34	5.1	461	6.2	0.8	0.6–1.1	0.3
Post term	27	4.1	322	4.4	0.9	0.6–1.4	0.7
High birth weight	35	5.3	845	11.4	0.4	0.3–0.6	< 0.01

**Table 3:** comparison of outcomes between early (age < 16 years) and late (age 17–19 years) adolescent deliveries

Outcomes	Early adolescent deliveries (n = 62)		Late adolescents deliveries (n = 600)		Odds ratio	95% CI	P value
	N	%	N	%			
LBW	14	22.6	100	16.7	1.5	0.7–2.8	0.3
Preterm delivery	12	19.4	69	11.5	1.9	0.9–3.7	0.07
Asphyxia at 1st minute	3	4.8	35	5.8	0.8	0.1–2.7	0.8

### Discussion

We have shown in this study that, 8.2% of deliveries at SAGHD are from adolescents with a significant decrease in the prevalence over a period of 6 years. Newborns from adolescent mothers were at higher risk of LBW, neonatal asphyxia and preterm delivery. There was no difference in the rates of occurrence of these outcome between early and late adolescents.

The prevalence of adolescent deliveries (8.2%) found among the study population fell within the 6–14% range reported in other studies in Sub-Saharan African countries [3, 16]. It is however slightly lower than that the prevalence recently observed in Buea (9.9%) and Bamenda (8.7%), both sub-urban settings in the country [13, 18]. It was also much lower than the prevalence of 20.4% obtained in a rural area in Cameroon [19]. The difference in the prevalence could be explained by our larger sample size and the social differences between the settings. Our study was carried out in an urban and cosmopolitan setting with higher level of employment and education. A higher prevalence (24.9%) was reported in northern regions of the country by Tebeu et al. in 2007 [16]. The northern part of the country is a rural, indigenous predominantly Muslim setting where early marriages are encouraged and education of the girl child is not prioritised. This could explain the higher prevalence in this setting. This marked difference in the prevalence of adolescent deliveries between muslim and non-muslim settings was also reported by Ezegwui et al. in Nigeria [20].

Our study showed that the contribution of adolescents to deliveries initially increased and then progressively decreased over time. This progressive decrease concur with the study carried out by Tebeu et al. in 2010 [21], and could be attributed to the decrease in the total fertility rate from 5.8 in 1991 to 5.0 in 2004 [21]. In addition, with the advent of HIV/AIDs, teaching on sexual education has increased in schools and on the media.

Many authors have reported an increase in the following adverse maternal and perinatal outcomes in adolescent pregnancies: preeclampsia/eclampsia, operative deliveries, maternal death, low birth weight, preterm deliveries, still births and neonatal asphyxia [12–14, 18, 19]. Our study showed that pregnant adolescents had higher rates of LBW infants, preterm delivery and neonates with low 5-min APGAR. Adolescents are still in a state of physical growth and adolescents who become pregnant during this phase of constant growth are predisposed to adverse neonatal outcomes [6], as they could compete with the developing fetus for nutrients leading to LBW neonates after delivery [15]. Also, most adolescents are single, unemployed and uneducated. Due to such financial constraints, adequate access to optimal prenatal care is usually not possible. Lack of appropriate prenatal care might lead to preterm deliveries [13].

In an attempt to determine whether pregnancy outcomes differed between early and late adolescents, a sub-group analysis was carried out. This analysis revealed no significant difference between the sub-groups. This is similar to results obtained by Njim and collaborators in Buea—a suburban setting [13]. Adolescents at all age groups therefore have equal risks of having adverse birth outcomes. This emphasises the need for policies to reduce the prevalence of adolescent pregnancy in our settings.

### Recommendations

In Cameroon the following programs—the National Population Policy in 1992, the “Maternal and Child Health Care and Family Planning Services Policy and Standards” in 1995, and the “Roadmap for Reduction of Maternal and Neonatal Mortality in Cameroon 2006–2015” [19, 22] have been instituted by the Ministry of Public Health to improve the reproductive health of Cameroonians without targeting special populations like adolescents directly. These policies need to be modified with adolescent-friendly targets and platforms to improve adolescent reproductive health and subsequently decrease the prevalence of adolescent pregnancies [19]. They should include increasing the number of health personnel specialised in adolescent health, increasing population health education awareness programs and promoting the use of adequate contraception amongst teenagers.

Indeed, studies have shown that the decrease in adolescent pregnancies and deliveries in some regions of Cameroon were accompanied by a decrease in overall perinatal complications in these settings like neonatal asphyxia [19]. In addition to instituting and reinforcing adolescent-friendly programs like contraception use which could reduce the prevalence of adolescent pregnancies and subsequently perinatal complications, emphasis should be placed on preconception care among this group which has been shown to decrease postnatal complications [23].

Improvements at service delivery level also should be made to improve the neonatal outcomes of adolescent pregnancies. These should include training healthcare personnel on basic neonatal resuscitation techniques, intermittent refresher courses for these personnel and improvement and creation of NICUs at tertiary and secondary levels, respectively.

### Conclusion

We found out in our study that, 8.2% of deliveries in the SAGHD are from adolescents, lower than what we recently found out in a sub-urban setting (9.9%). There was a progressive fall in the prevalence over the 6-year period. Adverse foetal outcomes attributed to adolescent deliveries were LBW, preterm delivery and low 1st minute APGAR. Measures directed towards the prevention of adolescent pregnancies should be implemented to reduce the associated neonatal morbidity and mortality.

## Limitations

We acknowledge the following limitations in our study: our study was carried out in 1 health facility in the Littoral region of Cameroon and so results may not be a true reflection of the situation in this urban area. In addition, our study being a retrospective study could have been subjected to the possibility of wrongly entered data. However, large sample sizes were obtained making the study powered enough to generate statistically and clinically relevant results.

## Additional files

**Additional file 1.** Reasons for exclusion of records from the study.

**Additional file 2.** Trend of adolescent delivery at SAGHD from 1st of January 2010 to 31st of December 2016. The x-axis shows the various years during the study was carried out. The y-axis depicts the prevalence of adolescent deliveries for each year. There was an initial rise in the prevalence from 2010 to 2011 then a progressive drop to 2015.

## Abbreviations

aOR: adjusted odd’s ratio
CI: confidence interval
HIV: human immunodeficiency virus
LBW: low birth weight
OR: odd’s ratio
US: United States
WHO: World Health Organisation
NICU: neonatal intensive care unit
