# Supplementary material for: Adolescent deliveries in urban Cameroon: a retrospective analysis of the prevalence, 6-year trend and adverse outcomes
Source: BMC Res Notes. 2018 Jul 13;11:469. doi: 10.1186/s13104-018-3578-0 (PMC6044024; doi:10.1186/s13104-018-3578-0)
Supplement: Supplementary file 1 — Additional file 1. Reasons for exclusion of records from the study. [file 13104_2018_3578_MOESM1_ESM.docx]

1091 records excluded because of incompletely filled files (maternal age, gestational age, type of delivery, birth weight and APGAR scores)

8911 records

605 records excluded because pages were torn off in the register

8306 records

10002 records

Final sample

8056 records

250 records excluded because

- Babies born before arrival to the hospital
- Records of multiple gestation
- Gestational age less than 28 weeks

**Figure S1: Exclusion of records in the retrospective phase**
