# Supplementary figures and images for: Adolescent deliveries in urban Cameroon: a retrospective analysis of the prevalence, 6-year trend and adverse outcomes
Source: BMC Res Notes. 2018 Jul 13;11:469. doi: 10.1186/s13104-018-3578-0 (PMC6044024; doi:10.1186/s13104-018-3578-0)

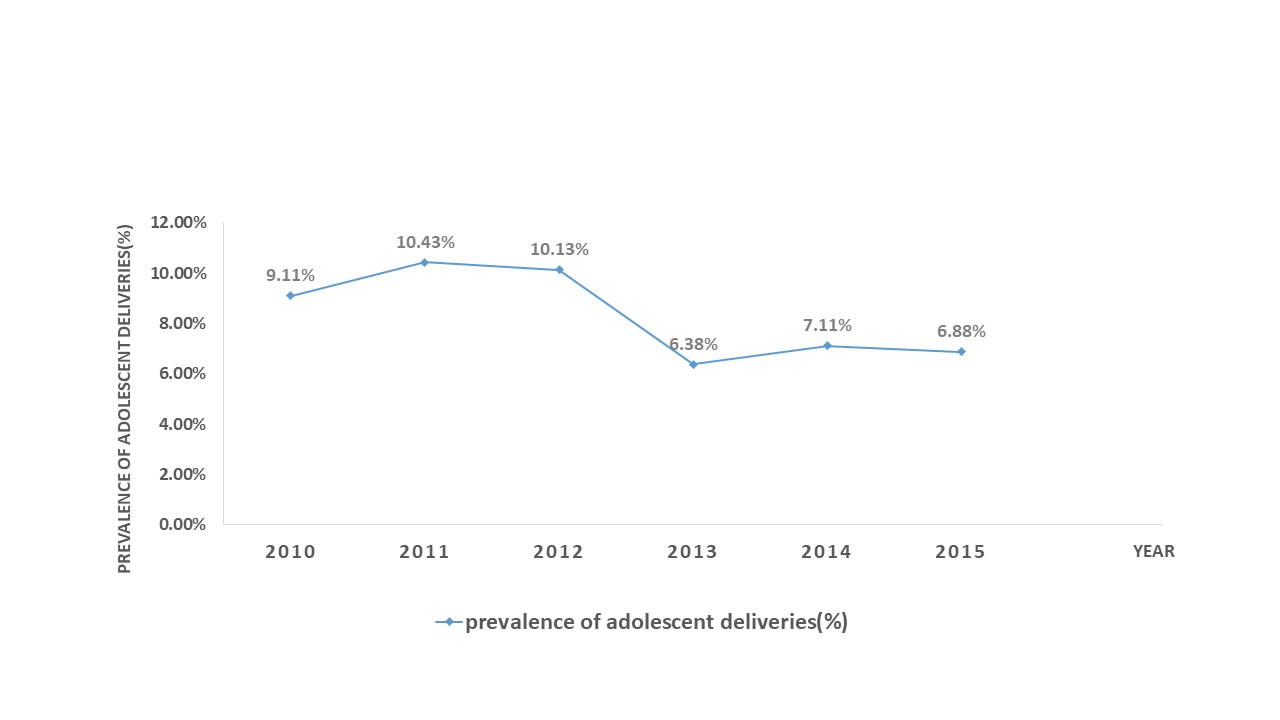

Supplement: Supplementary file 2 — Additional file 2. Trend of adolescent delivery at SAGHD from 1st of January 2010 to 31st of December 2016. The x-axis shows the various years during the study was carried out. The y-axis depicts the prevalence of adolescent deliveries for each year. There was an initial rise in the prevalence from 2010 to 2011 then a progressive drop to 2015. [file 13104_2018_3578_MOESM2_ESM.jpg]
